# Microbial assemblages in Arctic coastal thermokarst lakes and lagoons

**DOI:** 10.1093/femsec/fiae014

**Published:** 2024-02-02

**Authors:** Sizhong Yang, Xi Wen, Dirk Wagner, Jens Strauss, Jens Kallmeyer, Sara E Anthony, Susanne Liebner

**Affiliations:** GFZ German Research Centre for Geosciences, Helmholtz Centre Potsdam, Section Geomicrobiology, Telegrafenberg, Potsdam, Germany; Cyrosphere Research Station on the Qinghai-Tibet Plateau, State Key Laboratory of Cryospheric Science, Northwest Institute of Eco-Environment and Resources, Chinese Academy of Sciences, Donggang West Road 320, Lanzhou 730000, China; College of Electrical Engineering, Northwest Minzu University, Xibei Xincun 1, Lanzhou 730070, China; GFZ German Research Centre for Geosciences, Helmholtz Centre Potsdam, Section Geomicrobiology, Telegrafenberg, Potsdam, Germany; Institute of Geosciences, University of Potsdam, Karl-Liebknecht-Str. 24-25, 14476 Potsdam, Germany; Permafrost Research Section, Alfred Wegener Institute Helmholtz Centre for Polar and Marine Research, Telegrafenberg, Potsdam, Germany; GFZ German Research Centre for Geosciences, Helmholtz Centre Potsdam, Section Geomicrobiology, Telegrafenberg, Potsdam, Germany; Institute of Geology and Mineralogy, University of Cologne, Zülpicher Str. 49a, 50674 Cologne, Germany; GFZ German Research Centre for Geosciences, Helmholtz Centre Potsdam, Section Geomicrobiology, Telegrafenberg, Potsdam, Germany; University of Potsdam, Institute of Biochemistry and Biology, Karl-Liebknecht-Str. 24-25, 14476 Potsdam, Germany

**Keywords:** Arctic, coastal permafrost, microbial changes, thermokarst lagoon

## Abstract

Several studies have investigated changes in microbial community composition in thawing permafrost landscapes, but microbial assemblages in the transient ecosystems of the Arctic coastline remain poorly understood. Thermokarst lakes, abrupt permafrost thaw features, are widespread along the pan-Arctic coast and transform into thermokarst lagoons upon coastal erosion and sea-level rise. This study looks at the effect of marine water inundation (imposing a sulfate-rich, saline environment on top of former thermokarst lake sediments) on microbial community composition and the processes potentially driving microbial community assembly. In the uppermost lagoon sediment influenced from marine water inflow, the microbial structures were significantly different from those deeper in the lagoon sediment and from those of the lakes. In addition, they became more similar along depth compared with lake communities. At the same time, the diversity of core microbial consortia community decreased compared with the lake sediments. This work provides initial observational evidence that Arctic thermokarst lake to lagoon transitions do not only substantially alter microbial communities but also that this transition has a larger effect than permafrost thaw and lake formation history.

## Teaser

Transition from thermokarst lake to lagoon changed microbial assemblages more substantially than permafrost thaw and lake formation history.

## Introduction

Global climate warming is accelerating permafrost degradation. Gradual degradation is manifested by top-down permafrost thawing and thickening of the active layer. Thermokarst processes lead to rapid and deep thawing of permafrost and the development of thermokarst ponds and lakes, which is extremely common in ice- and organic-rich permafrost (Grosse et al. 2013, Olefeldt et al. 2016, Strauss et al. 2017). In Alaska, for example, thermokarst lakes have doubled in number and increased approximately by 37.5% in area from 1949 to 2009 (Walter Anthony et al. 2021). Thermokarst lakes in Siberian ice-rich permafrost have generally developed since the early Holocene (Jongejans et al. 2020). Arctic thermokarst lakes contribute to ∼80% of Arctic contemporary CH_4_ hotspot emissions and generally release large amounts of methane relative to CO_2_, and thus have a disproportionately high climate effect (Walter Anthony et al. 2018, 2021, Knoblauch et al. 2018).

Coastal erosion in the pan-Arctic can establish periodical or perennial connection of thermokarst lakes to the sea, which converts these lakes to lagoons. Thermokarst lagoons were estimated to account for 54% of the estimated total of ∼470 lagoons, which were identified along the Arctic coastline by remote sensing as of 2021 (Angelopoulos et al. 2021, Jenrich et al. 2021). Thermokarst lagoons represent a transitional state between freshwater thermokarst lakes and a fully marine environment. In these coastal lagoons, the hydrological connection to the sea plays a crucial role in facilitating the exchange of abiotic and biotic conditions between the two ecosystems (Gianuca et al. 2017). Vertical diffusion of marine water generates a sulfate-rich saline gradient on the top part of previous freshwater sediments (Schindler 2019). Along with the transition, microbial methane cycling community changes, for example, can influence carbon turnover and greenhouse gas emission (Yang et al. 2023). In an earlier study, we showed that within the sulfate zone, spatial co-occurrence of methane and sulfate thermodynamically favours sulfate-dependent anaerobic oxidation of methane, which mitigates methane emissions from thermokarst lagoons (Yang et al. 2023).

Thermokarst lakes and lagoons can serve as a natural laboratory to disentangle the mechanisms of microbial species replacement and evaluate the environmental controls on microbial community assemblage in rapidly degrading permafrost landscapes. Permafrost usually limits dispersal of species due to its frozen state (Bottos et al. 2018), while thawing will alleviate the dispersal constraints on microbes. The lateral and vertical expansion of thermokarst lakes presumably reworks the sediments to more homogeneous conditions than the previously frozen ground. The infiltration of saline marine water into the thawed sediment will not only rework the geochemical profile in the lake, but also introduce marine microbes to the newly formed lagoon ecosystems. Subsea permafrost was found to contain an enormous amount of organic carbon (Miesner et al. 2023) originating from onshore terrestrial permafrost, where microbial dynamics were found to be linked with changes of geochemical conditions along the sedimentation history (Mitzscherling et al. 2019). However, little is known about the changes of microbial structure and interspecies connection during the transition from thermokarst lakes to lagoons.

This study investigates how microbial communities, beyond those involved in methane cycling, shift along the transition from coastal thermokarst lakes to thermokarst lagoons in the Arctic. We presume that the restratification of geochemical profiles following thermokarst lake to lagoon transitions result in restructuring and convergence of the core consortia and address how microbial communities respond to the diverging geochemical conditions between thermokarst lakes and lagoons. We studied sediments of two thermokarst lakes and a lagoon from the Bykovsky Peninsula in northeastern Siberia where lagoons are extensively distributed and many thermokarst lagoons started to emerge about 2 ka before present (BP) (Jongejans et al. 2020) utilizing deep amplicon sequencing, and multiple numeric ecological approaches.

## Material and methods

### Study site and sampling

**Figure 1.**
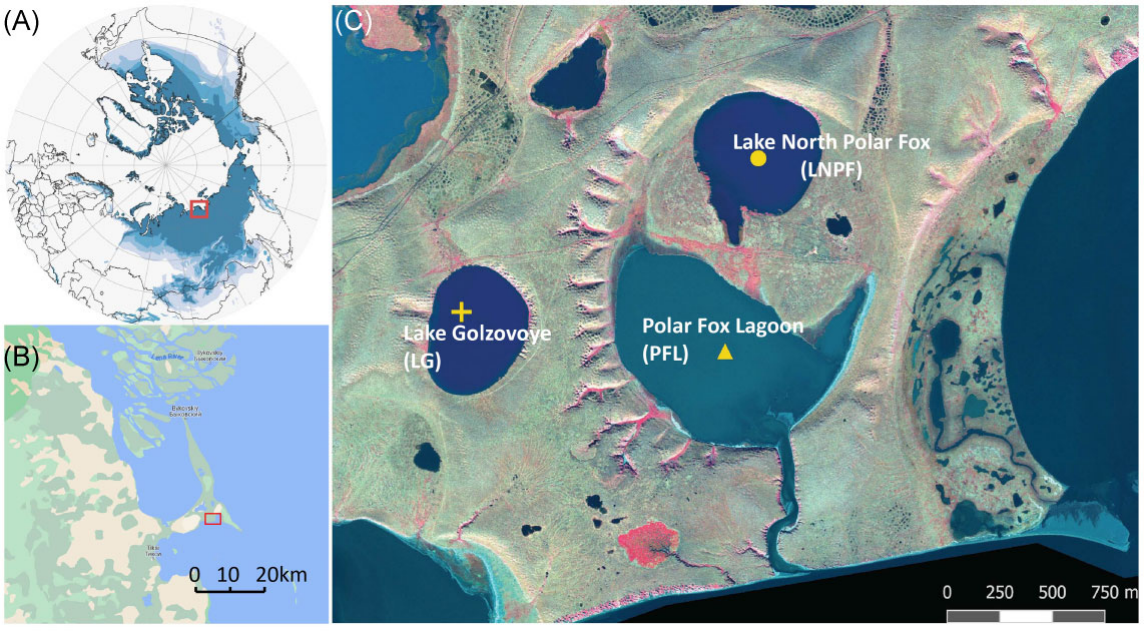
Maps of the study site showing (A) location with respect to the Northern Hemisphere and permafrost extent regions (B) location with respect to the Bykovsky Peninsula, and (C) relative location of the lakes and the lagoon (modified from Yang et al. 2023).

Sediment cores of three thermokarst bodies were retrieved on the Bykovsky Peninsula in the Laptev Sea, northeastern Siberian permafrost region. Lake Golzovoye (LG) and Northern Polar Fox Lake (LNPF) are freshwater thermokarst lakes while Polar Fox Lagoon (PFL) is a thermokarst lagoon to the south of LNPF (Fig. 1). Details about the three research sites can be found in Yang et al. (2023). Paleoclimatic proxies suggested thermokarst erosion to LG and LNPF since 8 cal ka BP and lagoon formation of PFL started about 2 cal ka BP (Jongejans et al. 2020). The PFL has more dynamic environmental conditions because of seasonal hydrological connection to Tiksi Bay, which is broken by ice in winter (Schirrmeister et al. 2018, Jenrich et al. 2021), while the thermokarst lakes maintain generally stable freshwater conditions.

Sampling and subsampling were performed during a field expedition in April 2017. Three cores (PG2420, PG2426, and PG2423) were retrieved for a total length of 5.2 m, 5.4 m, and 6.1 m, respectively, from sediments of lake LG, LNPF, and PFL, using an UWITEC piston corer. Subsequently, based on the specific research objectives of different participants during the joint field campaign, the core segments were either stored in N_2_-flushed, vacuum sealed bags at ∼4°C for pore-water analysis or sediment plugs were taken with sterile syringes directly in the field and subsequently frozen until further processing. The cores for microbial studies were divided into 49 samples, representing various depths in the sediment cores: 13 samples were retrieved from lake LG, 17 from LNPF, and 19 from PFL. In our recent study (Yang et al. 2023), we analyzed a subset of 23, which encompassed complete dataset of both geochemical and microbial information. In the current study, all the 49 microbial samples were used, independent of completeness of geochemical data, in order to obtain comprehensive information about microbial composition.

### Bulk parameters and pore water chemistry

Briefly, total carbon , total organic carbon, and total nitrogen were measured on bulk material using Elementar Micro Vario elemental analyzer (Elementar Analysensysteme, Hanau, Germany). The porewater was drained into a vacuum syringe in an anaerobic glove box (N_2_:H_2_, 95%:5%). The corresponding analyses included alkalinity, sulfate, chloride, nitrate, ferric, and ferrous iron. Alkalinity was measured by colorimetric titration, cations and anions were measured with suppressed ion chromatography, while the dissolved iron (ferric and ferrous) concentrations in pore water were measured via spectrophotometry by the ferrozine method (Viollier et al. 2000). All samples were measured in triplicates, the geochemical data together with detailed method description have been deposited at GFZ Data Services (https://doi.org/10.5880/GFZ.3.7.2022.001).

### DNA extraction and libraries preparation for Illumina sequencing

Total nucleic acids were extracted in duplicates using the PowerSoil-Kit (MO-Bio) according to the manufacturer’s protocol. Amplicon libraries were prepared by using barcoded primer pair sets (Uni515-F[5′-GTGTGYCAGCMGCCGCGGTAA-3′]/Uni806-R[5′-CCGGACTACNVGGGTWTCTAAT-3′]), with duplicates for each sample. PCR reactions (50 µl) contained 10× Pol Buffer C (Roboklon GmbH, Berlin, Germany), 25 mM MgCl_2_, 0.2 mM dNTP mix (ThermoFisher Scientific), 0.5 mM each primer (TIB Molbiol, Berlin, Germany), and 1.25 U of Optitaq Polymerase (Roboklon, Germany). The PCR program included an initial denaturation step at 95°C for 7 min, followed by 33 cycles at 95°C for 15 s, annealing at 60°C for 30 s, extension at 72°C for 30 s and a final extension step at 72°C for 5 min. After purification with the Agencourt AMPure XP kit (Beckman Coulter, Switzerland), the recovered PCR products were equilibrated into comparable equal amounts before pooling together with positive and negative controls. For the positive controls, we utilized a commercially available mock community (ZymoBIOMICS Microbial Community DNA Standard II). As for the negative controls, they consisted of the DNA extraction buffer and the PCR buffer. Sequencing was run in paired-end mode (2 × 300 bp) on Illumina MiSeq platform by Eurofins Scientific (Konstanz, Germany).

### Data processing, numeric, and statistical analysis

Raw sequences were demultiplexed by a custom Python script which used the ‘make.contigs’ function (pdiff=2, bdiff=1, other settings by default) in Mothur (v.1.39.5) (Schloss et al. 2009) to generate report files, upon which the raw sequences were demultiplexed into individual samples. After orientation correction with ‘extract_barcodes.py’ in QIIME1 (Caporaso et al. 2010), the sequences were processed by DADA2 (maxN=0, maxEE=2, truncQ=2, and minLen=175) and the output was reported in the format of an amplicon sequence variant (ASV) table (Callahan et al. 2016). The taxonomy was assigned against the SILVA138 database (Quast et al. 2013). Negative controls were employed to assess the contamination during DNA extraction and PCR processes, positive controls ensured that the sequencing itself did not introduce noticeable errors. Moreover, the sequencing duplicates demonstrated high consistency (Figure S1, Supporting Information). The contribution of different community members to the total abundance and beta diversity (Bray–Curtis dissimilarity, BC) was summarized by using *R* package otuSummary (version 0.1.1) (Yang 2020). The data obtained from each of the 49 samples, including their respective duplicates, were combined. The very rare ASVs with a cumulative count less than 10 across all samples were removed, resulting in the retention of a total of 25 880 ASVs. The microbial community dissimilarity was explored by nonmetric multidimensional scaling (NMDS) by using *R* package vegan (version 2.5.7) (Oksanen et al. 2019) based on the BC dissimilarity from Hellinger transformed data to mitigate the excessive effect of rare taxa. Following the clustering in NMDs, a hierarchical clustering (Figure S2, Supporting Information) was performed to identify the grouping feature of samples by *R* base package (R Core Team 2014). With that, permutational MANOVA was completed by ‘adonis2’ function of vegan package with BC matrix. To detect taxa, which were significantly enriched in the freshwater- and marine water-influenced sediments, linear discriminant analysis (LDA) effect size (LEfSe) was performed by using *R* package microbiomeMarker (v1.1.2), based on normalized data by using a negative binomial model (Cao 2021).

To detect associations between microorganisms from thermokarst lakes and lagoon, network analysis was implemented to explore the taxon co-occurrence patterns and the niche spaces. An initial filtering removed poorly represented ASVs with mean relative abundance < 0.5% from the whole community dataset, followed by a secondary filtering to get those ASV lineages with the Spearman correlation coefficient (absolute value > 0.75) and *P*-value (< .01). Afterwards, a network object was generated and analyzed by *R* package igraph (version 1.2.10) (Csardi and Nepusz 2006). Community modules of the network were detected with the ‘cluster_edge_betweenness’ algorithm of igraph package. The final network contained 194 ASVs. Based on the membership affiliation of each node (which represents individual ASVs), an NMDs plot was generated to explore the preferential occurrence of module members (ASVs) over different samples. A nonparametric Welch *t*-statistic was used to test the separation of each module over different groups with base package in R. In addition, the one-dimensional diagram was used to display the representative of individual modules over samples by using the function ‘*linestack*’ from vegan package.

## Results

### Environmental features

Exploratory ordination analysis on environmental variables, which were based on the porewater geochemistry and C, N content of bulk sediments, suggested that the marine water influenced samples, which were entirely composed of the uppermost 3 m sediments of PFL clustered away from the fresh water sediments (Figure S3, Supporting Information). The marine cluster were characterized by high levels of sulfate, salinity, and alkalinity, with highly enriched δ^13^C of methane (−54‰∼−37‰) in contrast to the depletion (−90‰∼−75‰) of freshwater sediment samples. The marine influence, thus, had a larger effect than that of the location.

### Community composition

**Figure 2.**
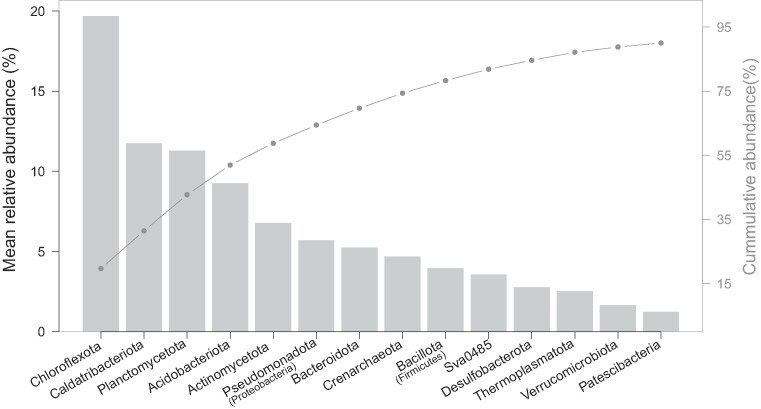
The abundance of dominant phyla with mean relative abundance greater than 1% over all samples. The 14 abundant phyla account for 90% of the total abundance. The *y*-axes in the left and right denote the scales for the barplot and cumulative abundance (line in grey), respectively.

**Figure 3.**
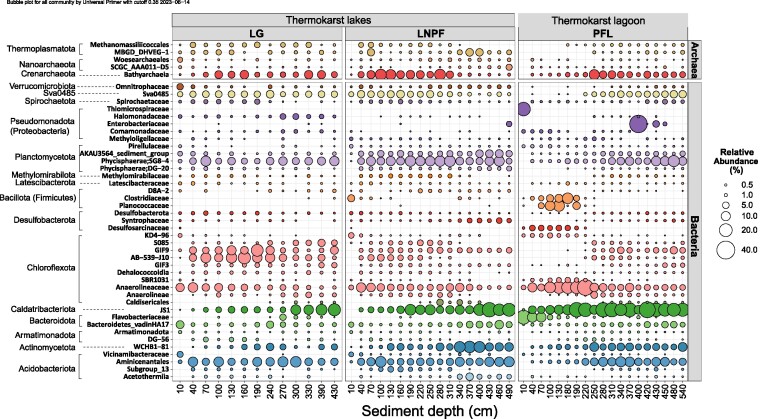
Bubble plot showing abundance variation of the 45 abundant lineages (with mean relative abundance > 0.35%) over depths for the three thermokarst lakes (LG: Lake Golzovoye, LNPF: Northern Polar Fox Lake, and PFL: Polar Fox Lagoon) in this study. Along the vertical axis, the taxonomy was presented at the family rank, and if assigning to the family level was not feasible, the next available higher taxonomic level was utilized. The relative abundance was calculated by combining the archaeal and bacterial ASVs and then collapsed at family level for this plot. The bubble colours correspond to different phyla, while the size of the bubbles reflects the average relative abundance.

The most abundant ASV lineage was Caldatribacteriota JS1, with a relative abundance of 9.7 ± 8.7% (mean ± SD) across all 49 samples. The predominant archaeal lineage (4.3 ± 3.7%) was affiliated to Bathyarchaeia within the phylum Crenarchaeota. At phylum level, a total of 14 taxonomic groups were identified with mean relative abundance > 1%, including 12 bacterial, and two archaeal phyla, which collectively account for 90% of the total abundance. Chloroflexota was the most abundant phylum (19.7 ± 8.5%), followed by Caldatribacteriota (former OP9, also known as Atribacteroita, 11.7 ± 8.3%) and Planctomycetota (11.3 ± 5.5%) (Fig. 2). The abundant archaeal phyla included Crenarchaeota (4.7 ± 3.7%) and Thermoplasmatota (2.5 ± 2.1%). Compositional variation over samples were displayed in detail at family level (Fig. 3), which also highlighted the distinct feature of the upper sediments of PFL.

Collectively, the 14 predominant phyla account for an average of 74% (first quantile: 66.2%, median: 75.1%, third quantile: 85.2%) to the total BC dissimilarity. The NMDs suggested two separate clusters of microbial communities, with one cluster consisting of samples from the brackish layer of PFL influenced by marine water (until the sample PFL_220 retrieved at depth of 220 cm), while the second cluster encompassed samples from freshwater sediments (Figure S3, Supporting Information). Interestingly, this pattern aligns closely with the two clusters observed in the environmental ordination, which correspond to sediments influenced by freshwater and brackish water, respectively (Figure S3, Supporting Information). The freshwater- and saltwater-influenced microbial clusters were statistically different (*P* < .001) according to adonis-based nonparametric MANOVA.

In the freshwater-influenced samples, a total of 8 characteristic taxa were observed with mean relative abundance > 2%, including Sva0485, Planctomycetota (AKAU3564 sediment group, SG8-4), Chloroflexota (e.g. GIF9, SCGC−AB−539−J10), Actinomycetota (WCHB1-81), Acidobacteriota (Aminicenantales), and Bathyarchaeia. These characteristic lineages occurred across most of the freshwater-influenced samples and their relative abundances are significantly higher than the marine-water influenced group. In contrast, the lagoon subgroup was represented by *Anaerolineaceae* (Chloroflexota), Sporosarcina, and *Clostridium sensu stricto* 13 (Bacillota, also known as Firmicutes). Additionally, lineages from Caldatribacteriota JS1 were abundant in both habitat groups. ANME-2a–2b was not highlighted as a characteristic lineage of the marine-water group as they largely prevailed only at the upper two layers among the total eight marine-water-influenced group, despite of their very high abundance in two sulfate-rich depths of lagoon sediments.

### Microbial co-occurrence and the environmental drivers

**Figure 4.**
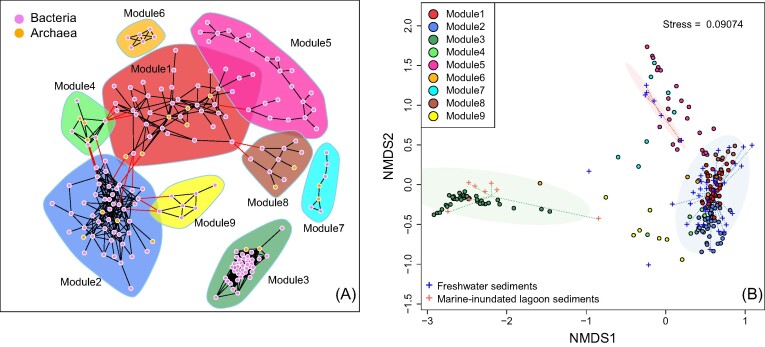
Network showing the pattern of module members (A). The ASVs in this plots were filtered out from the whole bacterial and archaeal dataset over 49 samples. The edges within a module and between modules were coloured in black and red, respectively. The NMDs (B) shows the association between module members (ASVs, represented by points) and samples (illustrated by cross symbols in the plot). The labels of the ASV lineage and samples were not shown in the plots to avoid crowdedness.

The network constituted 194 ASVs (diameter: 11.01673, mean distance: 4.688331, and average clustering coefficient transitivity is 0.765) with 912 edges, which show almost entirely positive association except for one negative interaction between ASV4 (Chloroflexota; GIF9) and ASV_193 (Actinomycetota; Cryobacterium). The network suggests nine nonrandom modules (modularity 0.5635) (Fig. 4). In this study, two modules (M1 and M2) exhibiting high species richness were predominantly observed in freshwater sediments, while a distinct and closely interconnected subgroup (M3) dominated the lagoon sediments influenced by marine water inundation (Fig. 4). The one-dimensional plot revealed that subgroups M3 and M6 were predominantly present in the brackish layers, whereas M7 was more commonly found in the upper layers. On the other hand, members of M1 and M5 were primarily abundant at the deeper part of freshwater sediments (Figure S4, Supporting Information).

**Figure 5.**
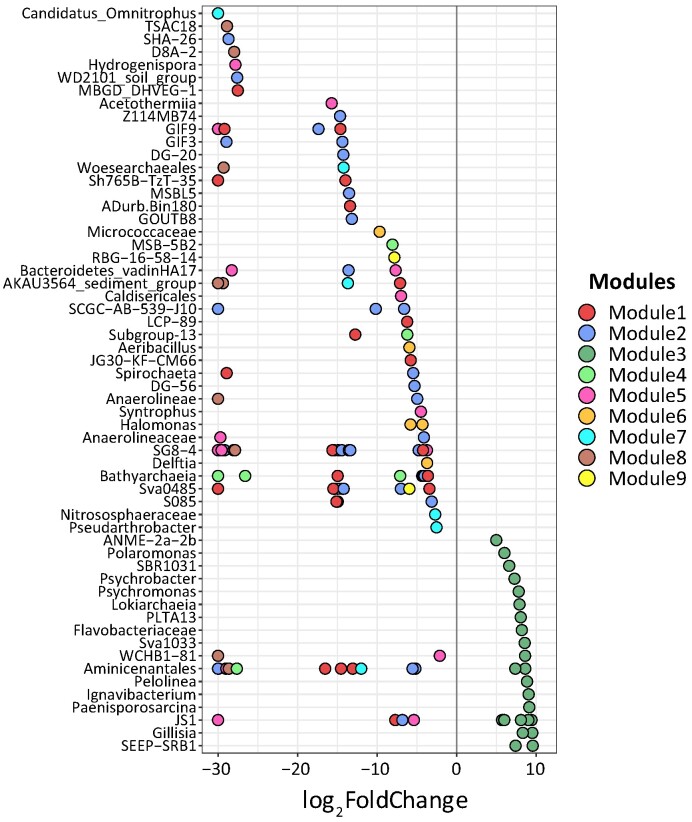
LDA effect size (LEfSe) identified characteristic taxa with statistically different abundance in the freshwater sediments and saltwater-influenced saline lagoon layers (the negative and positive parts along the *x*-axis, respectively). Taxonomy was given along the *y*-axis. If assignment to the genus level was not possible, the lowest possible taxonomic assignment was used. The dots in the plot were coloured by the modularity membership in the network plot Fig. 4, each point represents an ASV lineage.

**Figure 6.**
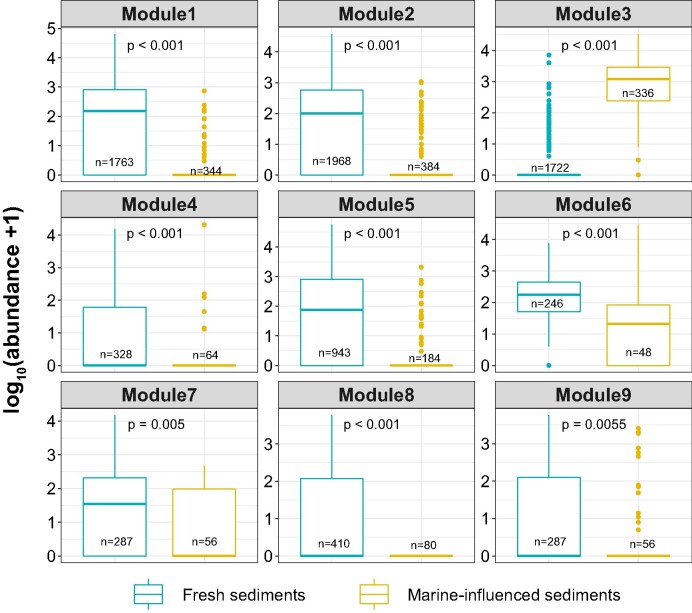
Boxplots comparing the means of freshwater sediments and marine-influenced lagoon sediment by using nonparametric Wilcoxon test for the nine modules identified from the network. For each module the number of ASV members were given in parenthesis. The abundance was log-transformed for better visualization, where log_10_(abundance +1) means transforming a value plus 1, which allows for handling values of zero in microbial community data. The number of observations was given within each boxplot. This plot was generated by R package ggpubr (version 0.4.0) (Kassambara 2020).

The module M3 comprised two archaeal and 40 bacterial ASVs, spanning across 10 different phyla. More than half of the ASV phylotypes were affiliated with Chloroflexota (11 ASVs, mainly from Anaerolineaceae), Caldatribacteriota (comprising eight ASVs of JS1), Pseudomonadota (also known as Proteobacteria, consisting of seven ASVs from Gammaproteobacteria in this study) and Bacteroidota (with six ASVs from Flavobacteriaceae and Ignavibacteriaceae). Additionally, this module included two archaeal lineages, namely from Halobacterota (one ASV from ANME-2a–2b) and Asgardarchaeota (one ASV from Lokiarchaeia). Such preference to marine-water inundation was also reflected by LEfSe analysis (Fig. 5). Nonparametric Wilcoxon test implied statistical significance of the abundance between freshwater sediments and marine-water influenced lagoons for each module (Fig. 6). For the freshwater sediments, pairwise *adonis* analysis did not reveal statistical significance across different modules.

## Discussion

This study demonstrates a substantial change in microbial communities following the infiltration of marine water into freshwater thermokarst lake sediments. These differences were greater than differences of microbial communities between the different lakes and the deeper (freshwater influenced) lagoon sediments. For the thermokarst lake sediments, multiple paleo-proxies have revealed relatively stable geochemical conditions with minor variations over about 8 ka BP when the studied upper 8 m of the sediments accumulated (Jongejans et al. 2020). In the thermokarst lagoon, marine-water inundation has generated a sulfate zone on top of the sediments since at least 2 ka BP. Both, the fresh- and marine-water-influenced sediments were probably subjected to relatively stable processes during the history of lake development, meaning that those geographically adjacent lakes have likely received pore waters from comparable sources and have experienced stable hydrologic conditions according to the low and stable electrical conductivity (Jongejans et al. 2020). Considering the longstanding anoxic and relatively stable conditions in the thermokarst lakes, the low level of environmental variability likely resulted in the overall convergence of microbial community composition. The thermokarst lagoon has seasonal connection with marine water, which not only caused more dynamic geochemical variation than the freshwater sediments, but also introduced new microorganisms. Owing to the periodic input of marine microorganisms, the seawater-affected part of the thermokarst lagoon sediment microbiome potentially experienced a greater influence of species gain or loss, in addition to the preceding effect of a homogeneous environment.

In our findings, we observe only slight differences in microbial community composition across the freshwater thermokarst sediments in general. This could potentially be attributed to the relatively shallow depth of the sediment profiles examined, the geographic proximity of the three research sites, and the relatively stable environmental conditions as mentioned above. The frozen conditions inherent to permafrost typically impose strong limitation on the spatial distribution and exchange of microbes, resulting in island biogeography patterns and divergent communities (Bottos et al. 2018), while the physical constraints within thermokarst sediments were greatly alleviated, which facilitates a higher turnover of species. Although spatial distance may still influence the rate of species replacement, the local and microspatial scales in thermokarst sediments are not expected to significantly impede the vertical and lateral exchange of microorganisms. This is especially true when there is a robust hydrological connection that facilitates species turnover within the sediments. The co-occurrence of closely related taxa, observed as module 3 in the thermokarst lagoon (Figs 4 and 5), further emphasizes the homogeneous nature of microbial communities in the sediments of all three research sites.

The shift from thermokarst lake (LNPF) to lagoon (PFL) resulted in a decreased diversity of the core microbial network (number of modules). This is manifested by the co-occurring bacterial subgroups that decreased from eight in freshwater sediments to one in the brackish lagoon sediments. In this study, almost all members within the different modules are positively connected to each other. Positive associations can enhance biological fitness of a module through mutualism or syntropy (Fisher et al. 2017), which often occurs in phylogenetically related microbes or is driven by similar environmental conditions and habitat niche (Weiss et al. 2016). Moreover, network modules were often regarded as a functional unit (Wang et al. 2017) and the multifunctional equivalent of trophic complementarity (Montoya et al. 2015). In this case, the overwhelming module diversity of freshwater sediments suggests higher functional diversity than the marine-water-inundated sediments. Since community modules are generally governed by habitat features and niche difference (Lima-Mendez et al. 2015), a substantial decline of module diversity in the brackish lagoon sediments may be a special adaptation to the sulfate-rich saline characteristics, which led to the observed distinct and densely clustered group separate from those of the freshwater sediments (Figure S4, Supporting Information). The distinct single module among the brackish lagoon group (M3) may represent a specialized functional group, which adapted to the sulfate-rich sediments. In line with the loss of module diversity of the network, a substantial decline in the representative taxa was also observed after the lagoon transition (Fig. 5; Figure S3, Supporting Information). The consistent change in microbial community assemblage provides evidence of significant habitat filtering following the thermokarst lake to lagoons transition.

Members of the representative module in the saline layers of the lagoon (M3), including ANME-2a–2b, Sva1033, Maribacter, Psychrobacter, and Lokiarchaeia, have potential roles as carbohydrate fermenters, reducers of sulfate, nitrate or iron, psychrophiles, or halophiles tolerant to cold environments (Table S1, Supporting Information). It is worth noting that ANME-2a–2b was particularly abundant in only two sulfate-rich sediment layers in the upper lagoon (not in the other six samples of the marine influenced module group), as highlighted previously (Yang et al. 2023). However, this lineage was not recognized as characteristic taxon because it was not abundant in most samples within a group. The anaerobic methanotrophs ANME-2a–2b engage in methane oxidation through syntrophic cooperation with sulfate-reducing bacteria (SRB), an essential process for reducing methane emissions from the ocean into the atmosphere (Boetius et al. 2000). The well-known (and potential) sulfate reducers such as Desulfobacterota SEEP-SRB1 and Sva1033 co-occurred with syntrophic partners, including members of Lokiarchaeia, Flavobacteriaceae, Caldatribacteriota JS1, Anaerolineaceae, and SBR1031, as such both parts can benefit from their establishment in the upper lagoon sediment layers. Additionally, prior research on the lagoon sediments, the thermokarst lagoon water column has been associated with strong methane oxidation during winter (Spangenberg et al. 2021).

Members of the bacterial JS1 group appeared to be very important overall. JS1 is affiliated to Caldatribacteriota (previous Atribacteriota, OP9) (Katayama et al. 2020), which was frequently observed abundant (31%–40%) in anoxic, organic-rich, and methane-containing bottom sediments (Webster et al. 2007, Carr et al. 2015, Lee et al. 2018), as well as in Arctic marine sediment with high methane concentrations (Carrier et al. 2020). A recent study on Baltic Sea methane hotspots suggested that JS1 together with *Dehalococcoidia* in Chloroflexi was strongly correlated with anaerobic methane oxidation rates (Iasakov et al. 2022). As such, the prevalence of bacterial phylotypes of JS1 in both marine and freshwater sediments of the studied sediments likely highlight the ecological importance of this generalist taxon. Aside from JS1, lineages of Bathyarcheota occurred as abundant archaeal members in the ecosystem. Bathyarchaeotal members are able to perform acetogenesis, potentially methane metabolism, and dissimilatory nitrogen and sulfur reduction, and can interact well with anaerobic methane-oxidizing archaea, acetoclastic methanogens, and heterotrophic bacteria (Zhou et al. 2019). The versatile metabolic potential of this lineage should facilitate their prevalence in anoxic sediments. Moreover, metagenomic data on the same lagoon studied here has recently explored nineteen Bathyarchaeotal genomes, which serve as peptide degraders and acetogenic microbes (Berben et al. 2022).

## Conclusion

This study represents an exploration of the microbial composition in Arctic coastal thermokarst lakes and a lagoon and suggests substantial shifts in microbial community due to brackish marine water inundation in the long term. It also demonstrated distinct microbial community compositions between marine- and fresh-water-influenced layers of the same thermokarst lagoon sediment representing former permafrost layers and newly formed lake sediment. This suggests that lagoon formation alters microbial assemblages more than thermokarst lake formation. In the uppermost lagoon sediment layers, microbial communities adapt to the sulfate-rich conditions with a reduction in spatial variation and diversity of the core microbial population. However, the sulfate-rich conditions in the top brackish layer of the thermokarst lagoon result in a distinct core species assemblage prevailing at the freshwater–marine interface.

## Supplementary Material

fiae014_Supplemental_File

## Data Availability

Sequencing data and the corresponding metadata are deposited at the European Nucleotide Archive (ENA) under BioProject accession number PRJEB49195, with samples accession number from ERS8483289 to ERS8483385.
