# Supplementary material for: Microbial assemblages in Arctic coastal thermokarst lakes and lagoons
Source: FEMS Microbiol Ecol. 2024 Feb 2;100(3):fiae014. doi: 10.1093/femsec/fiae014 (PMC10883142; doi:10.1093/femsec/fiae014)
Supplement: fiae014_Supplemental_File [file fiae014_supplemental_file.docx]

**Table S1**, Functional potential of ASVs within the single module of saltwater influenced lagoon sediments. Two ASVs which could be classified to class and order level were not included in this table.

| **ASVID** | **Lineages** | **Function or functional potential** | **References** |
| --- | --- | --- | --- |
| ASV_224, 246 | Aminicenantales | Polysarcchride fermenters, hydrogen provider | 10.1007/s00792-018-01073-5 |
| ASV_20, 93, 148, 168, 180, 203, 245, 264, 273 | Anaerolineaceae | Marine sediments, polysarcchride fermenters, hydrogen provider | 10.1002/9781118960608.fbm00301 |
| ASV_232 | ANME-2a-2b | Sulfate-dependent methane oxidizers | 10.1038/nature04617 |
| ASV_209 | Comamonadaceae (Polaromonas) | Psychrophiles, Anaerobic denitrifies and ferric iron reducing bacteria, hydrogen oxidizers | 10.1007/978-3-642-30197-1_238 |
| ASV_144, 192 | Desulfosarcinaceae (SEEP-SRB1) | Strict anaerobes, sulfate reducer prevailing in anoxic brackish water and marine habitats | 10.1002/9781118960608.fbm00329 |
| ASV_74, 76, 150, 195, 200 | Flavobacteriaceae (Maribacter) | Marine Clade of the Family Flavobacteriaceae, organic carbon remineralization | 10.1007/0-387-30747-8_26  10.1186/s12864-020-06971-7 |
| ASV_263 | Ignavibacteriaceae | Dissimilatory nitrate reduction to ammonium | 10.1038/s41396-021-01111-9 |
| ASV_49, 54, 55, 69, 84, 100, 105, 152 | Caldatribacteriota JS1 | H_2_ producers, fermenters using various substrates, improve growth of H_2_-scavenging taxa | 10.3389/fmicb.2018.02909  10.1038/s41467-020-20149-5 |
| ASV_174 | KD4-96 | Key acetogens | 10.1038/ismej.2014.148  10.1038/s41467-020-20149-5 |
| ASV_165 | Lokiarchaeia | Syntrophic association with sulfate-reducing and methanogenic partner | 10.1038/s41586-019-1916-6 |
| ASV_56 | Moraxellaceae (Psychrobacter) | Tolerant to low temperature and saline condition | 10.1093/femsec/fiy102 |
| ASV_63 | Planococcaceae (Paenisporosarcina) | Psychrophilic and halotolerant | 10.1128/JB.01472-12 |
| ASV_141 | PLTA13 | Potential in manganese oxidation | 10.1093/femsec/fiaa169  10.3389/feart.2020.586248 |
| ASV_137 | Pseudomonadaceae (Pseudomonas) | Facultative anaerobic denitrification | 10.1371/journal.pone.0118235 |
| ASV_103 | Psychromonadaceae (Psychromonas) | Halophilic and psychrophile, organic carbon, protein or lipid degraders | 10.1038/s41396-020-00817-6 |
| ASV_196 | Steroidobacteraceae | Functionally unknown, members containing CAZy family genes, might involve in carbohydrate degradation | http://www.cazy.org/b5699.html |
| ASV_107 | Sva1033 | Dissimilatory iron reduction | 10.1038/s41396-021-01014-9 |
| ASV_104 | WCHB1-81 | Potential microcystin (toxin) degradation | 10.1093/femsec/fiz162 |
| ASV_244 | SBR1031 | Carbohydrate (cellulose) fermentation | 10.1186/s13068-016-0524-z |


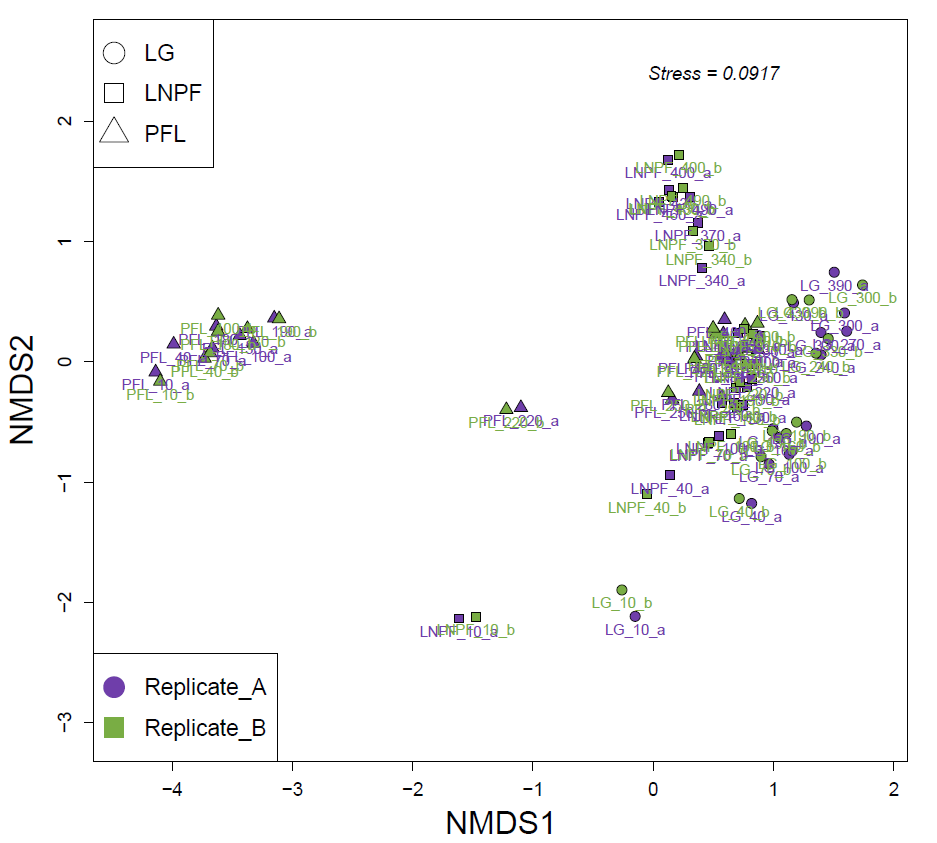


**Figure S1**, NMDs plot showing the robustness of the duplicates of each sample. Different shapes represent different thermokarst lake, and the two different colours denote the duplicates. LG: lake Golzvoye, LNPF: Lake North Polar Fox, PFL: Polar Fox Lagoon.


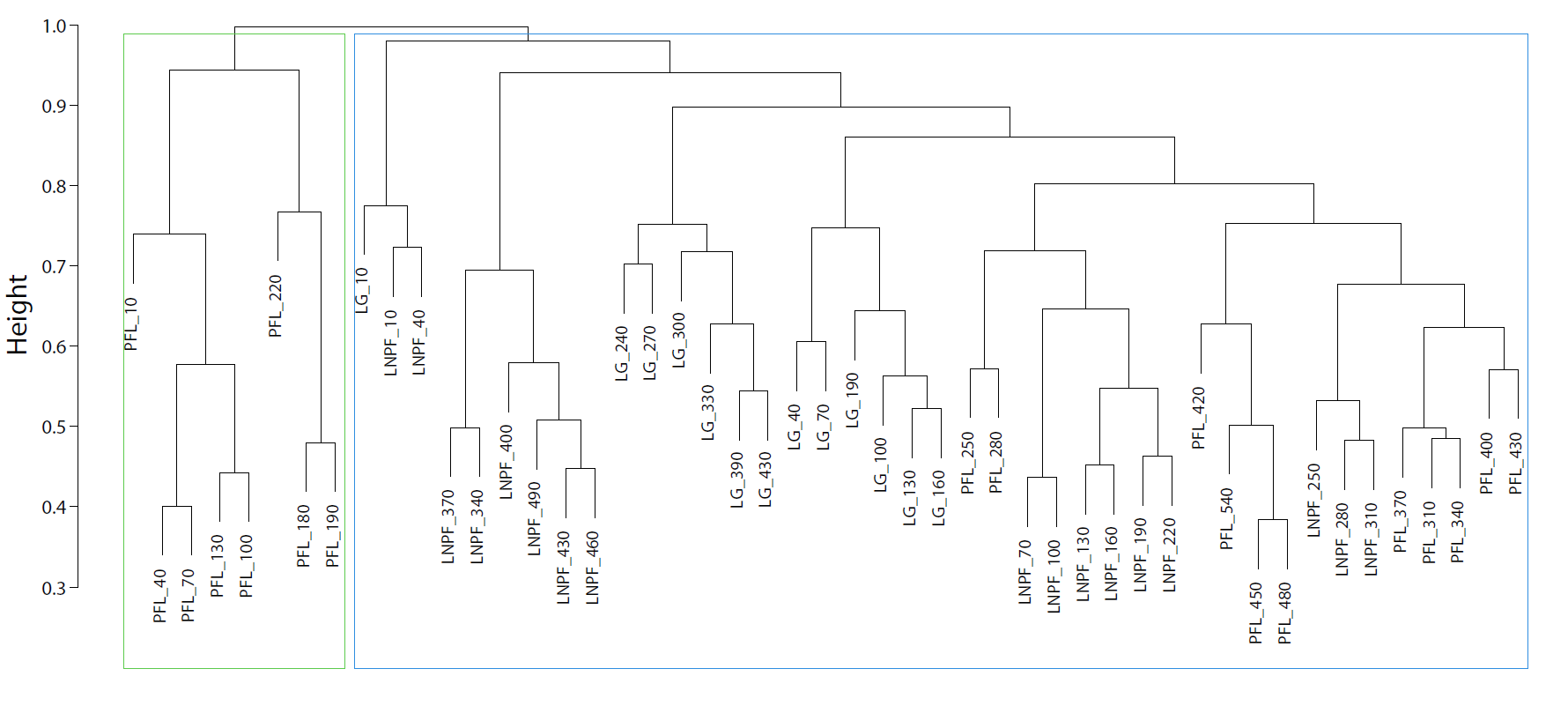


**Figure S2**, Hierarchical clustering suggested two distinct groups based on the Bray-Curtis dissimilarity of the microbial community. The group within the green rectangle consisted of 8 samples that are influenced by marine water, while the remaining samples within the blue box formed another group influenced by freshwater.


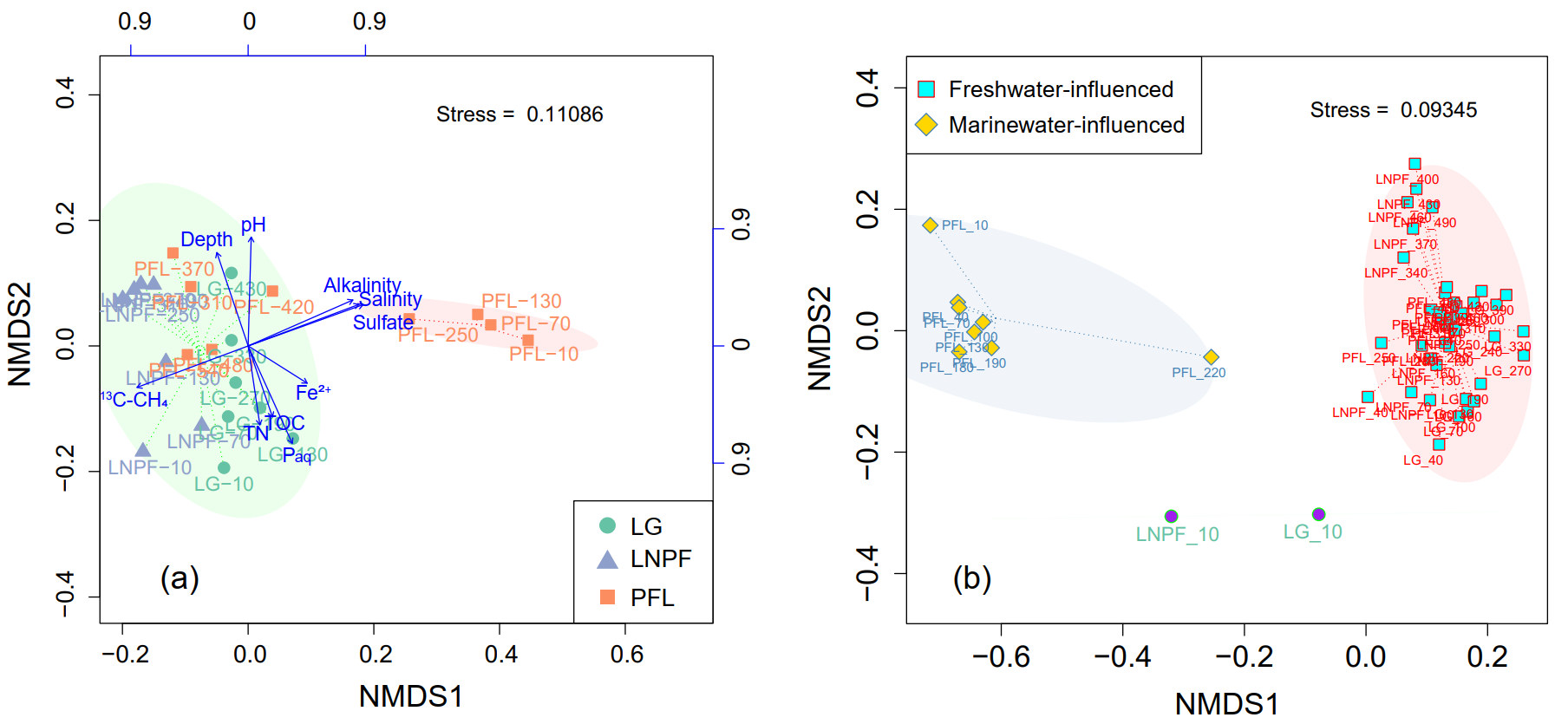


**Figure S3**, Non-metric multidimensional (NMD) scaling plots showing the two major clusters based on the environmental parameters (a) and microbial community dissimilarity (b). The ordination analysis was based on Bray-Curtis dissimilarity matrix. Figure S3a represents the ordination results for 23 samples with complete cases of all environmental variables, while Figure S3b corresponds to the ordination results based on the microbial community data from all 49 samples. Two outlier samples in Figure S3b were coloured differently. The environmental parameters with correlation *p*-value < 0.05 are displayed in this plot, providing an overview of the clustering patterns in the environmental composition. The ellipse shows 95% of the confidence regions for the locations of group centroids. The subgroup which is clustered in the right of the environmental ordination space is concert with the environmental separation in Figure 3a which was mainly represented by alkalinity, salinity and sulfate due to marine water inundation. LG: lake Golzvoye, LNPF: Lake North Polar Fox, PFL: Polar Fox Lagoon.


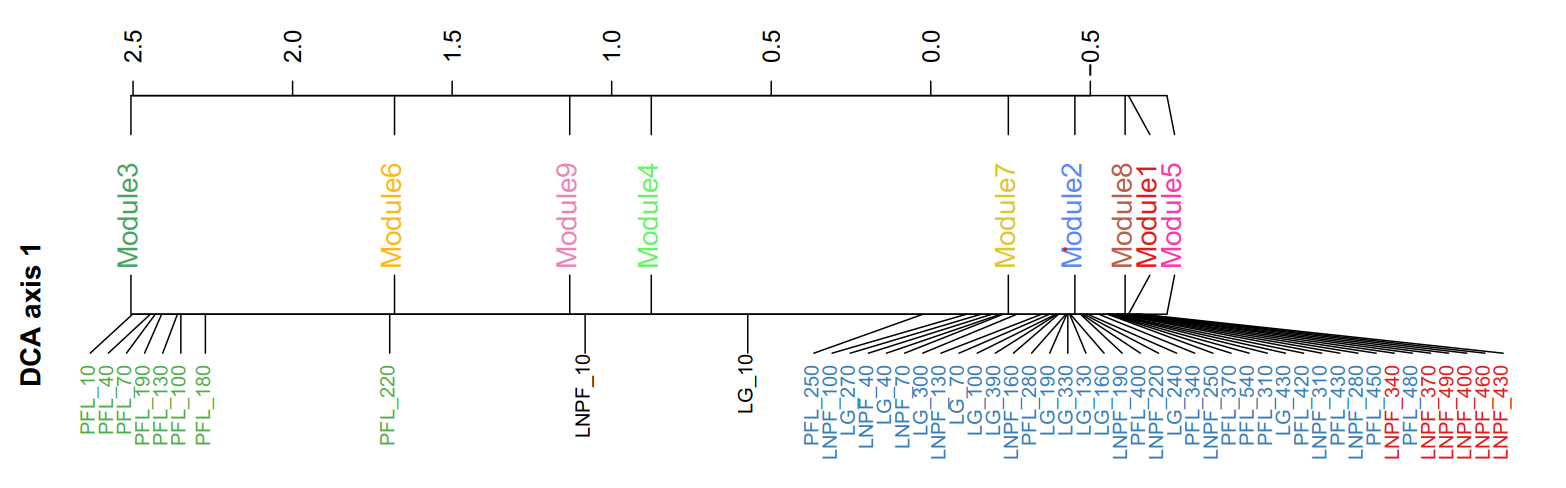


**Figure S4,** One-dimensional diagram showing the first vector of detrended correspondence analysis (DCA), where labels were shifted positions to avoid overwriting. The axes DCA1 and DCA2 represent 47.1% and 28% of the total variaiton of the subset of the modules community. Correlation plot shows that the axis DCA1 is largely associated with module 3, 6, 9 (which is prevalent in the upper 220cm of Polar Fox Lagoon) while the axis2 is more influenced by module 7 (which is more representative in the deep samples (340-490cm) of Lake North Polar Fox.
